# Microplastics in Female Reproductive and Pregnancy Organs: A Systematic Review

**DOI:** 10.3390/life16050746

**Published:** 2026-04-30

**Authors:** Bielka Carvajal, Rayen Vivero Sun, Francisca Piderit, Alejandra Zazueta, Cecilia V. Tapia, Martin Gotteland, Fabien Magne

**Affiliations:** 1 Department of Women’s and Newborn Health Promotion, Faculty of Medicine, University of Chile, Santiago 8380453, Chile; rayenvivero@uchile.cl; 2 Microbiology Interdisciplinary Core, Institute of Biomedical Sciences (ICBM), Faculty of Medicine, University of Chile, Santiago 8380453, Chile; francisca.piderit@ug.uchile.cl (F.P.); alezazueta28@gmail.com (A.Z.); 3 Medical Technology School, Universidad del Desarrollo–Alemana Clinic, Santiago 7610658, Chile; 4 Clinical Laboratory, Carabineros Hospital (HOSCAR), Santiago 7770201, Chile; cvtapiap@gmail.com; 5 Department of Nutrition, Faculty of Medicine, University of Chile, Santiago 8380453, Chile; mgottela@uchile.cl

**Keywords:** microplastics, women’s health, pregnancy, placenta, meconium

## Abstract

**Background:** Microplastics (MPs) have rapidly emerged as pervasive environmental contaminants with growing implications for human health. Evidence now shows that MP exposure may begin during pregnancy and extend into infancy. Foetal exposure to MP raises questions about MP presence within reproductive organs, maternal–foetal MP transfer and the potential impact of MP on women’s reproductive health. **Objectives:** To synthesise current evidence on the presence and distribution of microplastics in the female reproductive system and pregnancy-related organs. **Methods:** A systematic review of literature was conducted using Embase and Medline databases, supplemented by reference screening and manual searches. Studies were eligible if they examined MP in human female reproductive organs or pregnancy-related tissues and were published in English. **Results:** Eleven studies met the inclusion criteria. Across studies, MP detection varied substantially due to differences in sampling protocols, analytical techniques, and particle size detection thresholds. Cross-contamination and analytical method variability remained major methodological concerns. MP were consistently identified in follicular fluid, placental tissue, amniotic fluid, cord blood, and meconium. **Conclusions:** The presence of MP in both maternal and foetal compartments supports the possibility of in utero maternal-foetal MP transfer. A standardised protocol should be used to assess MP presence and MP’s impact on organs and tissues. The current variability of diagnostic tests, the lack of cofounding variables control and the reduced sample sizes limit the ability to determine how clinically relevant MP exposure is during pregnancy and to women’s reproductive health.

## 1. Introduction

Microplastics (MPs) have emerged as pervasive environmental contaminants, raising growing concern due to their persistence, ubiquity, and potential impact on human health [1]. MPs are typically defined as plastic fragments measuring between 1 µm and 5 mm in diameter. MPs have been found across environmental compartments, including freshwater and marine systems, soil, air, and indoor dust [2]. Major sources of MP pollution include plastic packaging, synthetic textiles, personal care products, industrial effluents, and the fragmentation of larger plastic debris [3,4,5,6,7,8]. Plastic packaging used for food and beverages represents a significant proportion of the MP we ingest daily [9]. MPs are considered persistent pollutants due to their capacity to undergo long-range atmospheric transport and accumulate in organisms over time. While most studies have focused on gastrointestinal and hepatic tissues, recent studies in humans have also found MP in the lungs, blood, brain, heart, bone, kidney, semen, spleen, and placenta [10].

Evidence from experimental studies and human biomonitoring indicates that MP have various harmful effects, including inducing oxidative stress, inflammation, endocrine disruption, impaired immunity, altered lipid and energy metabolism, neurological damage, and reproductive toxicity [11]. Several factors could influence MP’s impact on human health. For instance, particle size determines tissue penetration and cellular uptake, and MP shape may affect interactions with cells and biological barriers. The composition, electric charge and hydrophobicity of MP may influence their chemical reactivity and binding to biological molecules. All these characteristics together could modulate MP to trigger different or adverse biological responses.

Pregnancy is a vulnerable period, as maternal exposure to MP may compromise placental function and foetal development. MP in the placenta could disrupt immune development, organogenesis, and metabolic foetal programming. Early-life exposure to pollutants may have lasting consequences for infant health [12], potentially predisposing children to immune dysfunction, metabolic disorders, and other chronic conditions later in life [13].

In parallel, the rapid emergence of MP as a potential reproductive toxicant exposes a critical gap in the availability of standardised and clinically applicable diagnostic tools. Current detection methodologies (such as Raman spectroscopy, Fourier-transform infrared spectroscopy (FTIR), and pyrolysis–GC–MS) remain largely confined to research settings due to their high cost, labour-intensive workflow, and limited suitability for routine clinical application. Furthermore, the absence of validated biomarkers of exposure or biological effect restricts the capacity to identify at-risk women, monitor maternal–foetal transfer, or determine the clinical significance of MP accumulation.

Given accumulating evidence suggesting putative effects of microplastics on cells and tissues, this systematic review aimed to examine whether existing data support the presence of exposure levels relevant to women’s reproductive health, with particular attention to pregnancy as a vulnerable window. To address this aim, the review synthesised current evidence on the presence and distribution of microplastics in the female reproductive system and pregnancy-related organs, summarised reported associations with reproductive and neonatal outcomes where available, and identified methodological and diagnostic challenges that limit accurate exposure assessment, which were considered secondary objectives of the review.

## 2. Materials and Methods

A systematic review was conducted to explore the presence and impact of MP on female reproductive and pregnancy-related organs. The review protocol was registered in the International Prospective Register of Systematic Reviews (PROSPERO), under the registration number CRD42024584172. The review was conducted in accordance with the Preferred Reporting Items for Systematic Reviews and Meta-Analyses (PRISMA) guidelines.

Eligible studies were identified through an electronic search using the PICo (Population, Intervention, and Context) search strategy in April 2024. The databases used for the electronic search were Excerpta Medica Database (EMBASE) and the Medical Literature Analysis and Retrieval System Online (MEDLINE) through the OVID platform. Electronic searches encompassed all the available literature up to the date of the search. Details for each electronic search are provided in the Supplementary Materials. In addition to electronic searches, further studies were identified through the reference lists of included articles and a manual search conducted in August 2025. The manual search combined the keywords ‘microplastic’, ‘women OR woman’, and ‘reproductive OR reproduction’ using the Boolean ‘AND’.

The studies identified through the electronic searches were screened by two independent reviewers (BC-RV) in a two-step process using a screening tool (Supplementary Materials). In the first step, titles and abstracts were screened to determine whether the empirical studies addressed human health and the presence of microplastics. Later, the full texts of the articles that passed this first screening were carefully read to assess their eligibility using the screening tool. Disagreements between reviewers were solved by a third reviewer (FM). The screening process was managed using the Rayyan software. The same two-step screening process was followed for the studies identified through the reference lists and manual search. The reviewers’ agreement (BC-RV) was assessed using Cohen’s Kappa on a random sample of 200 articles (K = 0.93) before screening the entire set of articles identified through the electronic searches.

Studies were considered eligible for the review if they were

- Quantitative empirical studies;
- Focused on exploring the presence and impact of microplastics on women’s reproductive health, organs or during pregnancy;
- Were written in English.

Exclusion criteria considered:

- In vitro studies and studies conducted in animals;
- Studies focused on nano-plastics;
- Studies where tissues and organs were experimentally exposed to microplastics;
- Reviews, meta-analyses, comments, letters, protocols, and book chapters.

Data extraction from each included article was conducted independently by two reviewers using a standardised form (BC-FM). This form considered extracting the author’s name, the study country, the number and characteristics of the participants, the participants’ exclusion criteria, the methods used for MP detection, MP characteristics and concentrations across tissues, the cross-contamination protocol, and other relevant results obtained from the study. The data were entered into an Excel spreadsheet and validated by the research team. Findings from each included article were thematically grouped into categories related to the presence of MP in the placenta, female reproductive organs (such as the ovaries), and foetal tissues. Due to the diversity of MP detection methods, cross-contamination control protocols, and participants’ exclusion criteria across the included studies, the review findings are presented as a thematic description rather than in a data synthesis.

Two independent reviewers (BC-FM) assessed the quality of the included studies using the Critical Appraisal Skills Programme (CASP) checklist for descriptive/cross-sectional studies [14]. No article was excluded from the review based on its quality score.

## 3. Results

### 3.1. Study Selection

**Figure 1:**
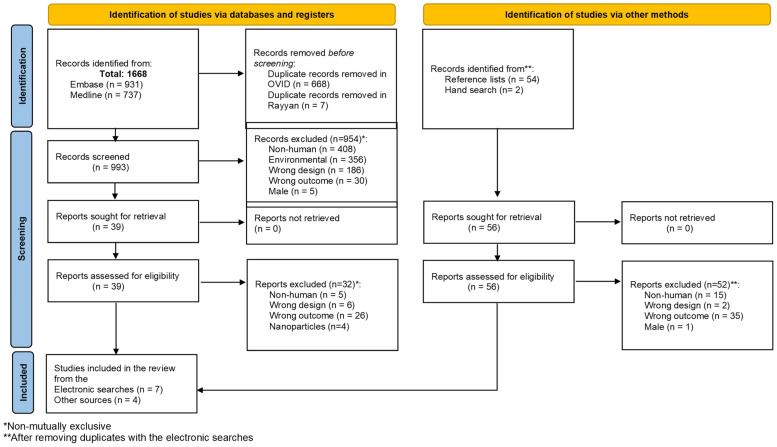
Prisma flow diagram.

The electronic searches across Embase and Medline yielded 1668 articles (Figure 1). After removing duplicates using the OVID platform, 1000 records were exported to Rayyan, where another seven articles were excluded as duplicates. The titles and abstracts of 993 articles were assessed for eligibility. Of these, 954 articles were excluded during the first-step screening because they did not meet the review’s inclusion criteria (Figure 1). After reading 39 full-text articles, 32 of them were excluded because they did not address the impact or presence of MP on human health (*n* = 26 + 5), were non-empirical studies (*n* = 6), or focused on nanoparticles (*n* = 4). The two-step screening process resulted in the inclusion of seven articles (Figure 1).

The reference list of all seven included articles was screened in two steps to identify eligible studies. The articles with titles suggesting they could be included in the review were added to a list, which was then compared against the results from the electronic search to identify potential duplicates. A total of 54 studies, not duplicated in the electronic search, were identified. After reviewing the full-text articles, 52 were excluded because they did not meet the review’s inclusion criteria (Figure 1). Two additional articles were included in the review through screening reference lists. After reviewing the first 10 result pages from Google Scholar, two additional articles were included in the review. Finally, 11 articles were included in the review. Despite the authors’ intent, most included articles focused on the presence of MP in female organs rather than assessing the impact of MP on women’s reproductive health.

### 3.2. Quality Appraisal

The quality of the eleven articles included in the review was assessed using the CASP checklist for descriptive/cross-sectional studies (Table 1). The decision to use the checklist for cross-sectional studies was made considering that all studies were observational, and in all cases, biological samples were tested only once over time (with no follow-up). The included studies were generally well conducted (Table 1). However, several articles that do not state that they are pilot studies do not report sample size estimates or provide any rationale for the number of participants chosen [15,16,17,18,19]. This issue makes it difficult to assess whether the findings of these studies are significant or representative. For instance, Amereh et al. compared MP levels in placentas from individuals with usual growth and those with intrauterine growth restriction (IUGR) [15]. However, no explanation is provided for the unbalanced group sizes (13 vs. 20) or for the sample’s statistical power. Additionally, although all studies collected data appropriately (Table 1; Q5), many failed to assess women’s exposure to MP or to measure cofounding variables exhaustively (such as environmental contamination, reproductive history, or current pregnancy details). Regarding the presentation of results, some graphs used make it difficult for the reader to examine MP concentration or qualities [17,20,21].

### 3.3. Description of the Studies

As described in Table 2, eleven articles published between 2021 and 2024 were included in the review. Five of the articles recruited participants from China [18,19,20,21,25], two from Germany [16,22], two from Italy [23,24], one from Iran [15], and one from the Czech Republic [17]. The included articles explored the presence of microplastics in the placenta [15,17,18,19,20,21,22,23,24,25], chorionic membrane [18,23], amniotic fluid [17,18], follicular fluid [16], and meconium [20,21,22,25]. One article examined the association between MP and preterm births [17], and another investigated it in relation to intrauterine growth restriction (IUGR) [15]. Both articles from Liu et al. belong to the same study [20,21].

MPs were detected through different methods (Table 3), including Laser Direct Infrared imaging [17,18,19,20]; Fourier transform infrared spectroscopy [22,26]; digital microspectroscopy [12,21]; Confocal Raman/spectrometry [25], and variable pressure scanning electron microscope coupled with transmission electron microscopy semi-quantitative analysis [24].

Lifestyle and MP environmental exposure questionnaires were used in three included studies [15,21,25]. In all three studies, custom-made questionnaires are used to assess women’s exposure to MP. Liu et al.’s study compared the abundance of MP in placentas and plastic use habits (never/seldom vs. often) [21]. After comparing groups, the authors found a statistically significant difference (*p* < 0.05) for the presence of polyethylene (PE) and using scrub cleanser and toothpaste (*p* = 0.005), total MP (*p* = 0.038) and polyamide (PA) presence (*p* = 0.038) and daily water intake, and the presence of polyurethane (PU) and drinking hydromel (*p* = 0.038) [21]. Besides eating and lifestyle habits, Zhu et al. also asked about air pollution sources near participants’ homes, the use of air purifiers, and the distance from major transport routes [25]. After analysis, Zhu et al. observed that women who drank tea ≥ 3 times/week had babies with a lower meconium MP load than women who drank tea < 3 times/week (*p* = 0.048) [25]. Other differences observed across groups regarding their eating habits, lifestyle and air pollution exposure were not statistically significant [25]. Amereh et al. examined the association between maternal habits and the presence of MP in the placenta using logistic regression analyses [15]. Drinking bottled water instead of boiled tap water (*p* = 0.01) and preferring takeaways over home-cooked meals (*p* = 0.02) were associated with a higher MP load in placentas [15].

Potential MP’s cross-contamination was controlled in all studies (see Table 3). Regarding the samples collected during deliveries, cross-contamination prevention measures included the use of cotton gloves and metal clamps for the umbilical cord [15,20,23,24]. In one study, amniotic fluid was obtained during amniocentesis using borosilicate syringes [17]. Two studies collected amniotic fluid using disposable syringes [18,25]. In one of these, authors assessed MP presence in a saline solution obtained using regular syringes and vacuum blood collection tubes as negative controls [18]. Regarding meconium, samples were usually collected by extracting the top portion of meconium directly from nappies/diapers [20,21,25]. Despite best efforts, MP contamination is challenging to control, as evidenced by negative control samples that test positive for MP [22].

The only study unrelated to pregnancy included in the review was that conducted by Grechi et al. [16] in Germany. These authors examined follicular fluid samples from individuals undergoing intracytoplasmic sperm injection. In their study, Grechi et al. found MP in all their samples of human follicular fluid (mean of 122.3 MP particles mL^−1^) [16] with an MP average size of around 11.5 ± 12.2 by 6.36 ± 5.1 μm [16]. Although a plastic-free protocol during analysis was used, no information was provided on how MP cross-contamination was controlled during follicle extraction and storage, especially considering that samples were initially retrieved for people’s personal purposes [16].

### 3.4. Microplastics in the Placenta

All studies included in the review confirmed the presence of MP in placental tissue [15,17,18,19,20,21,22,23,24,25] (see Table 2). Ragusa et al. (2021) were the first to describe MP presence in placentas from four of six women undergoing vaginal births [23]. Since then, MPs have also been detected at the core of the placenta from women who have undergone a caesarean delivery [22].

The number of MPs found in the placenta varies. Liu et al. (China) and Zhu et al. (China) reported an average of 18.0 MP/g [20,21] and 2.7 ± 2.65 MP/g [19], respectively. The Czech study reported values ranging from 0 to 10 MP per sample [17], and the Iranian study detected values ranging from 2 to 38 MP per placenta [22]. The remaining studies provide a qualitative account for the presence of MP in placentas [21,22,23,24].

Halfar et al. examined 10 placentas from women undergoing preterm labour (24 + 0 to 36 + 6 weeks of gestation), who also experienced preterm prelabour rupture of membranes (PPROM) [17]. MPs were found in 90% (*n* = 9) of placentas [17]. In one case, MP was detected only in the amniotic fluid, not in the placenta [17]. Although the authors take into account testing samples for possible cofounders of preterm birth, such as microbial invasion and inflammation markers (IL-6) [17], other risk factors, including women’s reproductive history, pregnant women’s health status, shortened cervical length, drug usage, and foetal conditions, are not accounted for in the study.

Amereh et al. reported that MP were present in 100% of placentas from women with IUGR pregnancies (*n* = 13), compared with 13.3% (4/30) of those from the control group [15]. In this study, placental MP content was found to correlate negatively with birth weight (r = −0.82, *p* < 0.001), neonatal length (r = −0.56, *p* < 0.001), and head circumference (r = −0.50, *p* = 0.001) [15]. As stated by the authors, the IUGR diagnosis was reportedly made by ultrasound, based on foetal biometry [15]. However, it is unclear when (pregnancy week) the IUGR diagnosis was made, and whether the professional who measured foetal biometry was the same for all women in the study. Likewise, no rationale is provided on how women in the control group were selected. All pregnant women recruited by Amereh et al. were initially described as healthy, and some relevant exclusion criteria related to IUGR were specified [15]. However, no further information on women’s reproductive history or clinical information about their current pregnancy was provided by the authors. Unlike in other studies, Amereh et al. accounted for women’s exposure to MP using a validated questionnaire. Unfortunately, the questionnaire was not included as Supplementary Materials [15].

Regarding the chemical identity of the MP detected in placenta in these studies, polyamide (PA) [18,19,20,21], polyurethane (PU) [18,20,21,22], polyethylene (PE) [15,17,20,21,22,25], polystyrene (PS) [15,19,22], and polypropylene (PP) [15,19,22,23], and, in lesser extent, polyvinyl chloride (PVC) [19] have been detected (see Table 2). According to Liu et al. [21], MP abundance in placentas was higher for women who used plastic bottles/cups (31.2 v 17.2 particles/g; *p* = 0.622) and for those who drank tap (61.5 v 19.2 particles/g; *p* = 0.264) or barreled water (31.2 v 20.4 particles/g; *p* = 0.620) compared to participants who seldom did these. Participants who frequently drank purified water exhibited a lower MP abundance (19.2 particles/g) compared to those who did not have this habit (26.8 particles/g) (*p* = 0.950) [21]. Unlike Amereh et al. [15], the questionnaire used by Liu et al. [21] to assess women’s use of plastic containers and consumption of plastic-packaged food in the previous year is available as Supplementary Materials, allowing other studies to use a similar approach and produce comparable data.

### 3.5. Placental Permeability to Microplastics: Emerging Evidence of Foetal Exposure

**Figure 2:**
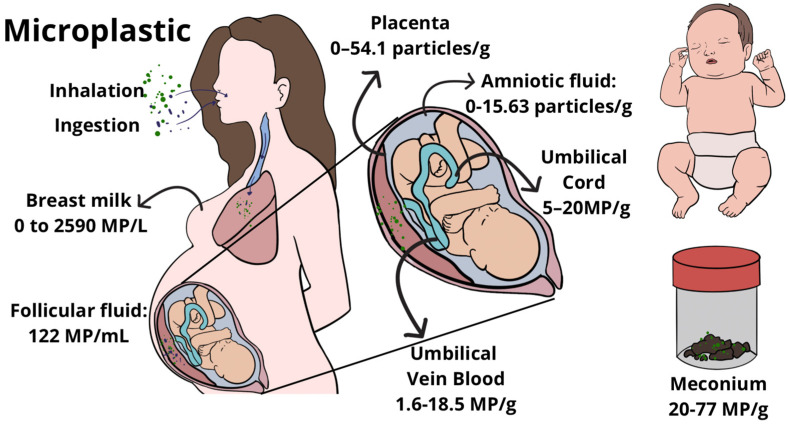
Microplastic Burden in Maternal, Foetal, and Neonatal Compartments.

Studies included in the review showed that MP were retrieved from the maternal [15,19,23] and foetal side of the placenta [15,19,20,21,23,25], at the core of the placenta [22], in the membranes [15,18,23], and within intracellular compartments such as the syncytiotrophoblast (Figure 2) [24]. These findings suggest not just surface contamination but actual tissue penetration.

The crossing of MP through the placenta is supported by the detection of MP in the meconium of neonates. Braun et al. attempted to establish a protocol for detecting MP that accounted for real-life clinical setting conditions [22]. For this, Braun and colleagues thoroughly sampled potential sources of MP within an operating theatre (including airborne fallout) and compared MP levels with those in placenta and meconium from two caesarean deliveries. Within their first attempt, Braun et al. detected MP presence (PE) in meconium, but the negative control turned positive for a different MP (PP). In their second attempt, the meconium sample remained positive for MP (PP, PS), while the negative control was negative [22]. Braun et al.’s study highlights the challenges of conducting MP research in real-world settings.

Regarding the distribution of MP in meconium, two studies suggest that meconium has a higher abundance of MP compared to placentas. Liu et al. found that MP abundance in the placenta (*n* = 18) was 18.0 particles/g, with PU, PA, and PE being the most frequently found MP [21]. In meconium samples (*n* = 12), MP abundance reached 54.1 particles/g, with the most frequent MP being PA, PU, and PVC [21]. Supporting this finding, Zhu et al. also reported that MP’s median abundance in meconium (20.75 particles/g) was higher than in the placenta (3.15 particles/g) and cord blood (1.1 particles/g) [25]. The higher abundance of MP in meconium compared to placentas in both studies could be explained by the accumulation of meconium in the human foetus’s intestine until after birth [20]. However, it is worth noting that in both Liu et al.’s [21] and Zhu et al.’s [25] studies, meconium was obtained directly from nappies/diapers, which could, in itself, represent a potential source of MP cross-contamination.

Besides meconium, MPs have also been found in amniotic fluid [17,18]. Halfar et al. collected amniotic fluid through amniocentesis using borosilicate glass syringes. Using this method, 6 of 10 amniotic fluid samples tested positive for MP or additives (range: 0–1.6 items/mL) [17]. Sun et al. collected 5 mL of amniotic fluid right after membranes had been broken and cord blood using disposable syringes from caesarean deliveries. Normal saline extracted with a disposable syringe and using vacuum blood collection tubes was used as a blank control in the study [18]. According to the authors, the two blank controls did not match the standard spectrum for more than 80%, concluding that these samples were free of MP [18]. Sun et al. found that MP’s abundance in amniotic fluid increased with maternal age (r = 0.64, *p* = 0.025) and body mass index before pregnancy (r = 0.59, *p* = 0.049) [18].

Similar to Sun et al.’s study, Zhu and colleagues used 5 mL disposable syringes to collect cord blood [25]. Of their three blank control samples, one was focused on cord blood collection and treatment. The authors report good-quality results for the placenta-faeces controls, but they do not report the results for the cord blood blank control [25]. MPs were detected in five out of nine cord blood samples in Zhu et al.’s study [25]. Sun et al. found an abundance of 2.726 (1.62–18.463) particle/g of MP in the umbilical blood vein [18]. More than 96% of MP found in the umbilical blood vein were between 20 and 100 μm [18].

Figure 2 summarises literature-reported observations across studies included in this review and is intended to illustrate general trends rather than precise quantitative values.

## 4. Discussion

### 4.1. Summary of Key Findings

The articles included in the review show that MPs are detected in follicular fluid and pregnancy-related organs and tissues, supporting the hypothesis of transplacental passage, and consistently indicate that the foetus is exposed to MPs (Figure 2). The consequences of MP exposure on human foetal development are not yet clear, as the included studies have relatively small sample sizes, are non-randomised, and do not fully control for cofounding variables associated with adverse neonatal outcomes. Likewise, at the moment, the review cannot determine a cutting point or limit to suggest that MP presence is harmful to women’s reproductive organs or the foetus.

Animal and human data suggest potential associations between MP exposure and adverse developmental outcomes. Exposing mouse dams to 5 μm polystyrene resulted in a reduction in foetal size of up to 12% at the highest MP concentration (106 ng/L) [26]. In this review, one study showed MP load was negatively associated with the weight, length, and head circumference of newborns born to healthy mothers [15]. Together, these findings suggest that prenatal MP exposure may influence foetal growth, although causal relationships in humans remain to be established.

### 4.2. Early-Life Exposure and Developmental Programming

The effects of MP exposure during the utero-foetal period may persist into the neonatal stage, particularly through breastfeeding and formula feeding. A positive dose-dependent relationship has been observed, with higher amounts of breast milk consumed by infants being associated with higher MP levels in the babies’ faeces [21]. The preparation of infant formula, particularly when using hot water for sterilisation or mixing, can release millions of PP particles into the formula consumed by infants [21,27]. Repeated sterilisation, boiling, or exposure to alcohol, steam, or UV light further increases MP shedding from bottle surfaces [21]. A positive association has been documented between the frequency of bottle use, the volume of formula consumed, and the abundance of MP detected in infant faeces [21].

Zhang et al. detected higher concentrations of PET in meconium and infant faeces compared to adult faeces [28]. These findings highlight the heightened exposure of infants to MP due to chronic ingestion, which may trigger localised gut inflammatory responses and, if MP translocates across the intestinal barrier, potentially lead to systemic effects. The Developmental Origins of Health and Disease (DOHaD) paradigm posits that the intrauterine and early postnatal environment critically shapes long-term health outcomes [29]. In this regard, adverse conditions caused by exposure to MP during pregnancy, which is a critical window of development, could induce adaptive physiological and metabolic changes in the foetus. These adaptations, while beneficial for short-term survival, may lead to permanent alterations in organ structure and function, thereby increasing susceptibility to chronic diseases later in life. Given that low birth weight is a well-established risk factor for adverse health outcomes throughout life for increased mortality, prediabetes, diabetes, and high blood pressure, further studies examining correlations between MP exposure and adverse neonatal outcomes are warranted [30,31].

### 4.3. Biological Mechanisms Underlying Microplastic Translocation and Foetal Exposure

Several studies included in this review suggest plausible biological mechanisms underlying MP-associated effects. One study indicated MP presence could increase syncytiotrophoblast cellular stress [24]. The administration of polystyrene MP in pregnant mice altered the uterine blood supply and immune balance, characterised by reduced arterioles and decidual NK cells, increased helper T cells, a shift toward M2-dominant macrophages, and an overall immunosuppressive cytokine profile [32]. Experimental evidence further supports the capacity of MP to cross the placental barrier. Furer et al. estimated the crossing of MP (PS) using model human in vitro bio-barrier models by cultivating a tight and functional placental monolayer on a nanofiber membrane. They observed a 5.5 ± 0.3% translocation of the applied MP dose after 6 h of placental perfusion [33]. Other studies, using similar-sized MP (PS), reported translocation of 30% and 11% of particles after 3 h and 6 h of transfusion, respectively [34,35]. Together, these studies showed that MP could pass through the placenta. Discrepant permeability levels observed between the two studies could be due to differences in the commercial sources or batches with distinct physicochemical properties, as well as in the perfusion system and protocols (e.g., perfusion medium, exposure concentrations, flow rates, tubing length) [36]. Animal and human in vitro/ex vivo studies evidence that various types of environmental particles, including micro/nanoplastics, particulate matter, and engineered nanomaterials, such as gold (Au), silica (SiO_2_), silver (Ag), and titanium oxide (TiO_2_), can cross pregnancy barriers and reach foetal tissues [22,24,36]. Nevertheless, further research is warranted to delineate the extent and clinical implications of this permeability, as well as to elucidate the underlying biological mechanisms facilitating the translocation of microplastics across the placental barrier.

### 4.4. Positioning of the Findings Within the Current Literature

One of the strengths of the review is that we employed multiple strategies to identify eligible studies and achieved high agreement among reviewers before conducting the screening. Additionally, the research team possesses a broad set of skills to understand the impact of MP on women’s reproductive health. Two other systematic reviews on topics similar to ours have been published since our electronic search. The review ‘Impact of microplastics on pregnancy and foetal development’ included 12 articles, of which eight were shared with our review [37]. About the remaining four articles of Sharma et al.’s review, two [38,39] of them would not have been included in our review due to not being focused on women’s reproductive or pregnancy organs, and the third article was published after the electronic search for our review was conducted [40]. The fourth one was not identified by our literature or manual search [41]. This study retrospectively examined placentas collected in Hawaii that were not collected in accordance with a plastic-free protocol [41]. Wiengrill et al.’s findings show a positive trend of MP accumulation in placentas over 15 years [41]. Moreover, 60% of the examined placentas tested positive for MP in 2006, 90% in 2013, and 100% in 2021 [41]. Zurub et al.’s study examined placentas from vaginal (*n* = 5) and caesarean (*n* = 5) deliveries and found that all placenta regions tested positive for MP, regardless of delivery mode [40]. The second review that is somehow similar to ours is Inam’s review on the ‘impact of microplastics on female reproductive health: insights from animal and human experimental studies’ [42]. However, as clearly described in the title, Inam’s review considers data from female animals and women, whereas we focused only on women’s data. Fifteen articles are included in Inam’s review [42]. Of the total studies, eight considered human samples, with the majority (*n* = 5/8) included in our review. Human samples in Inam’s review included: human serum, placenta, breastmilk, follicular fluid, and human blood [42].

### 4.5. Methodological Heterogeneity and Current Limitations

Despite growing evidence of MP presence in obstetric tissues, there is still no standardised, validated, or clinically applicable method for detecting MP in obstetric tissues, leading to considerable variability in reported abundances, polymer types, and particle sizes. This heterogeneity is mainly driven by differences in sampling strategies, extraction protocols, and analytical platforms. Indeed, differences in reported MP abundances across studies largely reflect the analytical technique used rather than actual environmental concentrations (Table 4). Techniques such as Raman microscopy, micro-FTIR, LDIR, SEM or pyrolysis-GC/MS each provide complementary but incomplete diagnostic information, varying in their ability to detect small particles, identify polymer composition, or preserve morphology. Critically, most methods struggle to detect particles < 1 µm, which likely represent the fraction with the highest biological reactivity. These methodological discrepancies hinder exposure quantification and prevent the definition of clinically meaningful thresholds or toxic doses. Contamination control represents another key diagnostic challenge. Despite the use of non-plastic protocols, MP can enter samples from airborne dust, laboratory materials, collection devices, or diapers used for meconium sampling. At the same time, harsh digestion steps may degrade more labile polymers, leading to underestimation. These opposing biases underscore the urgent need for standardised, contamination-controlled protocols and harmonised analytical workflows. Together, these limitations emphasise that the field currently lacks the diagnostic infrastructure necessary to generate reliable exposure assessments, compare findings across studies, or translate MP quantification into clinically relevant biomarkers for maternal–foetal health.

## 5. Conclusions

Current evidence demonstrates the presence of microplastics in female reproductive and pregnancy-related tissues, including follicular fluid, placenta, amniotic fluid, and foetal compartments such as cord blood and meconium. Emerging data further suggest associations between microplastic exposure and adverse pregnancy outcomes. However, progress in understanding maternal–foetal microplastic transfer and its biological consequences is fundamentally constrained by the lack of standardised, sensitive, and contamination-controlled detection methods. This methodological heterogeneity represents the principal factor limiting mechanistic insight, exposure quantification, and cross-study comparability. Overcoming this limitation through the development and validation of harmonised analytical workflows and clinically applicable biomarkers is therefore the critical next step to enable robust risk assessment and to inform evidence-based public health and clinical guidance on microplastic exposure during pregnancy.

## Figures and Tables

**Table 1:** Quality appraisal using CASP for descriptive/cross-sectional studies.

Author (Year)	Q1	Q2	Q3	Q4	Q5	Q6	Q7	Q8	Q9	Q10	Q11
Amereh F. (2022) [15]	Y	Y	N	Y	Y	N	Y	N	Y	Y	Y
Braun T. (2021) [22]	Y	Y	Y	Y	Y	Y	Y	Y	Y	Y	Y
Grechi N. (2023) [16]	Y	Y	Y	Y	Y	N	Y	Y	Y	Y	Y
Halfar J. (2023) [17]	Y	Y	Y	Y	Y	N	Y	Y	Y	Y	Y
Liu S. (2023a) [20]	Y	Y	Y	Y	Y	Y	Y	Y	Y	Y	Y
Liu S. (2023b) [21]	Y	Y	Y	Y	Y	Y	Y	Y	Y	Y	Y
Ragusa A. (2021) [23]	Y	Y	Y	Y	Y	Y	Y	Y	Y	Y	Y
Ragusa A. (2022) [24]	Y	Y	Y	Y	Y	CT	Y	Y	Y	Y	Y

**Table 2:** Description of studies.

Study	Country	Study Design ^1^	Aim ^1^	Exclusion Criteria	Tissue	MP Detection	Particle Detected ^2^	Main Finding
Amereh et al. (2022) [15]	Iran	Not mentioned (comparison of groups)	To report the uptake of plastic particulates in the placentas of pregnant women and their association with reduced foetal growth in IUGR ^3^ pregnancies.	Several exclusion criteria were applied, including cancer status, medicine intake affecting intestinal reabsorption, alcohol use, smoking, advanced diabetes, poor weight gain, high blood pressure, heart disease, intrauterine infections, kidney or lung diseases, malnutrition, anaemia, sickle cell anaemia, and autoimmune disease.	Placenta (*n* = 43; 13 were IUGR pregnancies)	13% of the control group	PE (67.7%), PS (33.3%)	Inverse associations between MP presence and birth weight (r = −0.82, *p* < 0.001), length (r = −0.56, *p* < 0.001), head circumference (r = −0.50, *p* = 0.001), and 1 min Apgar score (r = −0.75, *p* < 0.001) among the IUGR group, compared to those that were nominated as normal pregnancies
						100% of IUGR pregnancies	PE (43%), PS (36.4%), PET (14.9%), PP (5.6%)	
Braun et al. (2021) [22]	Germany	Pilot observational study	To establish a protocol for the detection of MPs in human placenta and foetal meconium.	Not mentioned.	Placenta ^4^ (*n* = 2)	100% of placenta (1 negative control tested positive)	Patient 1: PE, PP, PU. Patient 2: PP, PS	Human placenta and meconium samples tested positive for MP. Controlling MP contamination is crucial for accurate MP detection.
					Meconium (*n* = 2)	100% of meconium (1 negative control tested positive)	Patient 1: PE, PP. Patient 2: PP, PS	
Grechi et al. (2023) [16]	Germany	Not mentioned (cross-sectional)	To assess the extent to which MPs might be bio-accumulating in the follicular fluid of women.	Not mentioned.	Human follicular fluid (*n* = 7)	100% of samples	PVC, PE, PS, PP, PU, RUB, and ABS.	MPs are found in the follicular fluid of women.
Halfar et al. (2023) [17]	Czech Republic	Cohort study	To investigate the occurrence of microplastic particles and additives in both human amniotic fluid and placentas.	Not mentioned. Samples were tested for Ureaplasma species, Mycoplasma hominis, and Chlamydia trachomatis.	Placenta from preterm births (*n* = 10; 0.5 g each)	80% of samples	CPE, PE-HD, Zinc calcium PVC stabiliser, others	In 9 of 10 patients, MP or additives were detected in amniotic fluid, the placenta, or both.
					Amniotic fluid (PROM) ^5^ (*n* = 10)	60% of samples	CPE, PE-HD, PET, PTT, paper-coated plastic, others	
Liu et al. (2023a) ^6^ [20]	China	Pilot prospective study (6-month follow-up)	To detect the MP in placentas and meconium samples and explore the potential association of MP exposure with microbiota in placentas and meconium.	HIV, gastrointestinal disease, cancer, and other severe pathologies.	Meconium ^7^ (*n* = 12)	100% of samples	PA (60.22%), PU (22.58%), PTFE (4.06%), PVC (2.99%) and others	Sixteen types of MP were identified in all matrices. MP detected in samples with a size of 20−50 μm was more than 76.46%.
					Placenta ^8^ (*n* = 18)	100% of placentas	PA (50%), PU (28.75%), PE (11.01%), PET (2.74%) and others	
Liu et al. (2023b) ^6^ [21]	China	Pilot prospective study (6-month follow-up)	To assess MP’s exposure in placenta, meconium, infant faeces, breast milk, and infant formula samples, and assess the potential sources of pregnancy and lactational exposure to MP.	HIV, gastrointestinal disease, cancer, and other severe pathologies.	Meconium (*n* = 12)	100% of samples	PA (60.22%), PU (22.58%), PTFE (4.06%), PVC (2.99%), Others	Sixteen MP types were identified, and >74% of the MPs were 20–50 μm in size. Water intake and the use of scrub cleanser or toothpaste may be sources of exposure for pregnant women.
					Placenta (*n* = 18)	100% of placentas	PA (50.09%), PU (28.7%), PE (11.01%), PET (2.74%), Others (10%)	
Ragusa et al. (2021) [23]	Italy	Pilot observational descriptive preclinical study	To explore MP’s presence in the human placenta.	Several exclusion criteria were applied, including gastrointestinal disease, cancer, organ transplantation, HIV and other severe pathologies; alcohol abuse, smoking, certain diets, medication, and dental treatments close to the delivery.	Placenta ^8^ and chorioamniotic membranes (*n* = 6)	66% (*n* = 4)	PP (4/12), n.d. for 8 samples	12 MP fragments with spherical or irregular shapes were found in 4 placentas. All MPs were pigmented.
Ragusa et al. (2022) [24]	Italy	Not mentioned (cross-sectional)	To locate MP within the intra/extracellular compartments in human placenta and to understand whether their presence and location are associated with possible structural changes in cell organelles.	Several exclusion criteria were applied, including particular diets, diarrhoea or constipation in the two weeks before childbirth, antibiotic use, medication use interfering with intestinal absorption, diagnosis of a gastrointestinal disease, cancer, organ transplant, HIV, any other disease that requires medical treatment, dental treatments, and alcohol abuse.	Placenta ^4,8^ (*n* = 10)	100% of placentas	Not specified	Presence of fragments compatible with MP in the cellular compartment of the human placenta.
Sun et al. (2024) [18]	China	Prospective study	To identify MP in maternal blood, foetal appendages, and umbilical vein blood, and analyse the association between MP in maternal blood and those in foetal appendages and umbilical vein blood.	As per Ragusa et al. (2021) [23]	Women’s venous blood ^6^ (*n* = 12)	8.176 particles/g	PA, ACR, PU, FKM	MP presence in the umbilical cord, maternal blood, foetal membrane, amniotic fluid, placenta and umbilical vein blood. >90% of MP measured between 20 and 100 μm in diameter. MP abundance in amniotic fluid increased with maternal age (R = 0.64, *p* = 0.025) and body mass index before pregnancy (r = 0.59, *p* = 0.049).
					Amniotic fluid (*n* = 12)	4.795 particles/g	PA, PU, PMMA, PET, FKM	
					Umbilical vein blood ^6^ (*n* = 12)	2.726 particles/g	PU, CPE, PA	
					Foetal membranes (*n* = 12)	6.561 particles/g	PA, PU, PMMA, ACR, CPE, PE	
					Placenta (*n* = 12)	4.675 particles/g	PU, PA, ACR, CPE	
					Umbilical cord (*n* = 12)	10.397 particles/g	PU, PA, CPE, ACR	
Zhu et al. (2024) [25]	China	Pilot study	To investigate the exposure to MP in mothers and infants and to explore the potential sources of MP contamination in the placenta, cord blood, and meconium.	Heart and chronic kidney disease, psychiatric disorders, hypertension, diabetes, gestational hypertension, and gestational diabetes.	Placenta (*n* = 9)	100% of placentas	CEL 23.5% (8/34), PNB 17.6% (6/34)	>80.47% of MPs detected in samples had a size of 100–400 μm. The load of MP is higher in meconium than in the placenta and in the cord blood. The abundance of MP in meconium from women who drank tea ≥ 3 times/week during pregnancy was lower than in those who drank less (*p* = 0.048).
					Cord blood ^9^ (*n* = 9)	55% of cord blood samples	PB 42.9% (6/14)	
					Meconium ^7^ (*n* = 9)	100% of meconium	PE 38.8% (31/80), Blend 32.5% (26/80) and PCL 7.5% (6/80)	
Zhu et al. (2023) [19]	China	Not mentioned (cross-sectional)	To evaluate the presence and characteristics of microplastics in 17 placentas	Not mentioned.	Placenta (*n* = 17)	100% of placentas	PVC (47.8%), PP (14.55%), PBS (10.90%), PET (7.27%), PC (6.91%), PS (5.82%), PA (5.45%), polyester fibre (2.91%), PE (1.45%), PAM (0.73%), and PSF (0.73%)	Microplastics were detected in all placenta samples, with an average abundance of 2.70 ± 2.65 particles/g. The MP ranged in size from 20.34 to 307.29 μm, and most (80.29%) were smaller than 100 μm.

^1^ As stated by the authors; ^2^ Polymers abbreviation: PE (Polyethylene), PS (Polystyrene), PET (Polyethylene terephthalate), PP (Polypropylene), PVC (Polyvinyl chloride), PU (Polyurethane), ABS (Acrylonitrile Butadiene Styrene), PTFE (Polytetrafluoroethylene), PA (Polyamide/Nylon), PMMA (Polymethylmethacrylate), ACR (Acrylates), PNB (Polynorbornene), PC (Polycarbonate), PCL (Polycaprolactone), CEL (MP cellulose), CPE (Chlorinated PE), PTT (Polytrimethylene terephthalate), RUB (Rubber), PAM (Polyamide), PSF (Polysulfone), FKM (Fluororubber), PB (Poly butene isotactic); ^3^ Intrauterine growth restriction; ^4^ From caesarean sections; ^5^ Preterm prelabour rupture of membranes; ^6^ Both articles belong to one study; ^7^ Collected directly from nappies/diapers; ^8^ From vaginal deliveries; ^9^ Collected using a disposable syringe.

**Table 3:** Comparative overview of sampling approaches and analytical detection parameters reported in microplastic studies.

Study	Sample Type(s)	Sampling Method and Setting	Sampling Material in Contact with the Sample	Contamination Control Measures	Analytical Method	Microplastic Size Range
Amereh et al. (2022) [15]	Placenta	Collected at delivery from 43 women; IUGR vs. normal pregnancies	Cotton gloves with inner rubber gloves; metal clippers; pre-washed cotton towels; glass labware	Plastic-free protocol during delivery and lab work; reagents filtered through 0.22 µm filters; plastic-free lab coats; cotton gloves; three ultrapure-water QC samples	Digital microscopy + Raman microspectroscopy	Most particles < 10 µm
Braun et al. (2021) [22]	Placenta, Stool, Meconium	Collected during caesarean sections; placenta cut into blocks outside OR under sterile bench; meconium and stool transferred with a metal spatula into glass bottles.	Cleaned glass bottles, metal spatulas, stainless-steel filters, Aqua Kem Blue solution	All glassware rinsed with ultra-pure water, covered with aluminium foil, oven-dried; negative controls using instruments without tissues; extensive contamination controls, including operating theatre materials and airborne fallout	FTIR ^1^ microspectroscopy in transmission after digestion	MPs > 50 µm
Grechi et al. (2023) [16]	Follicular fluid	Follicular fluid aspirated during clinical/abattoir procedures	Glassware where possible; aspiration materials new/sterile	All procedures in a laminar flow hood; all apparatus rinsed 3× with 0.1 µm filtered ultra-pure water; all reagents and water filtered (0.1 µm); vials closed with aluminium foil; procedure blanks with aspiration materials.	MP isolation followed by spectroscopic identification.	Not explicitly stated
Halfar et al. (2023) [17]	Amniotic fluid, Placenta	Clinical collection from 10 patients with PPROM; paired AF and placenta	Glass fibre filters, metal tools, and glassware only	Filtered reagents (≤1 µm), glass fibre filters, metal tools, glassware, lab cleaning, cotton cloths, aluminium foil wrapping; 6 airborne blanks; fibres excluded from analysis.	FTIR-ATR ^2^ after KOH digestion	10–50 µm
Liu et al. (2023a,b) [20,21]	Placenta, Meconium	Placentas and meconium from 18 mother–infant pairs	Stainless steel (13 µm) membranes; highly reflective glass slides; glass utensils	All reagents vacuum-filtered through 13 µm stainless steel; glassware rinsed with ethanol; procedural blanks with ethanol; recovery tests for several polymers (PP, PE, PS, PET 91–97%; PVC, PU 84–87%)	LDIR ^3^ (Agilent 8700)	20–500 µm
Ragusa et al. (2021) [23]	Placenta	Six placentas from physiological pregnancies collected at delivery; internal parenchymal portions sampled	Metallic scalpels; metallic containers; glass sample containers (no plastic gaskets)	Plastic-free preparation; substitution of plastic tools with metallic or glass; cotton gloves; cold-chain in metal/glass	Raman microspectroscopy after digestion and filtration	5–10 µm identified
Ragusa et al. (2022) [24]	Placenta	Ten placentas collected at delivery; intraparenchymal samples (5 mm) taken by a pathologist using metallic tools	Metallic scalpel; metallic containers; glass containers for storage; plastic-free preparation.	Plastic-free procedures; replacement of plastic tools with metal/glass throughout EM preparation.	VP-SEM and TEM ^4^ for localisation of fragments compatible with MPs	Sub-micron to micron fragments visualised; numerical detection limit not specified
Sun et al. (2024) [18]	Maternal blood, Placenta, Amniotic fluid, Foetal membrane, Umbilical cord, Umbilical vein blood	12 caesarean sections; 3 g tissue per foetal appendage; blood via venepuncture/syringe	Glass bottles, glass beakers, glass sample bottles; Pasteur pipettes; cotton laboratory coats; nitrile gloves; activated-carbon masks	“No plastic” principle; reagents filtered three times; glassware rinsed with ethanol; saline blanks mimicking blood/AF collection with syringes and tubes; LDIR analysis of two blanks (no MPs >80% match)	LDIR	20–100 µm
Zhu et al. (2024) [25]	Placenta, Cord blood, Meconium	After delivery: cord blood via sterile disposable syringe into a glass tube; placenta pieces in a sterile stainless-steel container; meconium swabbed from the diaper surface with wooden cotton swabs.	Non-plastic consumables for sampling: glass anticoagulant tubes, stainless steel containers, wooden swabs, specimen boxes pre-treated with tinfoil.	All reagents filtered through 1 µm glass fibre membranes; non-plastic tools for sampling and digestion; three procedural blanks for each matrix; recovery tests for PE, PS, PVC (80–110%)	Micro-Raman spectroscopy	100–400 µm

^1^ Fourier transform infrared spectroscopy; ^2^ Attenuated Total Reflectance; ^3^ Laser direct infrared; ^4^ Variable pressure scanning electron microscope with transmission electron microscopy.

**Table 4:** Comparative overview of spectroscopic and imaging techniques used for microplastic characterisation in biological matrices.

Technique	Main Strengths	Key Limitations (for MPs in Bio-Matrices)	Approximate Practical Size Range for Particles ^1^
LDIR imaging [43,44,45,46]	Rapid, automated infrared imaging using a quantum cascade laser, offering high throughput and reasonable polymer specificity.	Limited spectral range (~975–1800 cm^−1^) compared with full FTIR; requires reflective substrates (e.g., MirrIR or gold-coated filters) due to reflectance geometry; reduced sensitivity for very small particles because of diffraction and substrate texture; and still undergoing standardisation and inter-laboratory validation.	Reliable for MPs ≥ 20 µm; 5–20 µm detectable on smooth reflective substrates, with reduced performance on textured filters.
FTIR–ATR [47,48] (Attenuated Total Reflectance)	Minimal spectral preparation; excellent spectral quality and polymer specificity; widely available for biofluid analysis.	Contact-based technique requiring individual particle contact with the crystal, low-throughput for complex matrices, limited penetration depth (few µm), primarily applicable to larger particles or additives after digestion.	Effective mainly for MPs ≥ 100–300 µm; smaller particles produce weak or mixed spectra.
FTIR [22,47,49,50] microspectroscopy (µFTIR, transmisión)	Automated/semi-automated whole-filter imaging with size, count, morphology, and polymer identification; widely used in biota and clinical samples.	Requires IR-transparent non-polymer filters, limited spectral range and spatial resolution, long acquisition times and no mass estimation.	Reliable mainly for irregular MPs ≥ 50 µm, with poor performance for 10–50 µm fragments and fibres
Micro-Raman microspectroscopy [22,47]	Higher spatial resolution than FTIR, analysis of smaller particles, strong polymer/additive discrimination, suitable for coloured and transparent MPs.	Limited field of view, long mapping times, fluorescence interference, and high operator expertise requirements.	Enables routine analysis of MPs below 10 µm and outperforms FTIR for particles < 50 µm.
Electron microscopy [43,47] (VP-SEM/TEM)	Very high spatial resolution and ultrastructural detail; TEM enables nanometre-scale localisation, and VP-SEM mitigates charging in biological samples.	Morphological only without EDX, no polymer identification, labour-intensive TEM preparation with potential artefacts, and not quantitative for polymer type or mass.	Imaging from tens of nanometres to >10 µm; well suited for <1 µm MP-compatible fragments with complementary chemical identification.

^1^ Size ranges are indicative of practical detection capabilities reported in environmental and clinical microplastic analyses and should not be interpreted as absolute physical thresholds.

## Data Availability

The original contributions presented in the study are included in the article/Supplementary Materials, further inquiries can be directed to the corresponding author.

## Supplementary Materials

The following supporting information can be downloaded at: https://www.mdpi.com/article/10.3390/life16050746/s1, S1: Electronic searches per database; S2: Screening tool; S3: CASP checklist for descriptive/cross-sectional studies. Reference [51] is cited in the supplementary materials.
