# Supplementary material for: Microplastics in Female Reproductive and Pregnancy Organs: A Systematic Review
Source: Life (Basel). 2026 Apr 30;16(5):746. doi: 10.3390/life16050746 (PMC13208567; doi:10.3390/life16050746)
Supplement: Supplementary file 1 [file life-16-00746-s001.zip › Supplementary material_Jan26.pdf]

Supplementary material 1: Electronic searches per database

Embase 1974 to 2024 via OVID 13 April 2024

Searches	Results	Type
1	microplastic pollution/ or microplastic/	9567
2	(women or woman).mp. [mp=title, abstract, heading word, drug trade name, original title, device manufacturer, drug manufacturer, device trade name, keyword heading word, floating subheading word, candidate term word]	1991414
3	(people or person*).mp. [mp=title, abstract, heading word, drug trade name, original title, device manufacturer, drug manufacturer, device trade name, keyword heading word, floating subheading word, candidate term word]	2663907
4	(reproduction or reproductive).mp. [mp=title, abstract, heading word, drug trade name, original title, device manufacturer, drug manufacturer, device trade name, keyword heading word, floating subheading word, candidate term word]	456696
5	microplastic*.mp. [mp=title, abstract, heading word, drug trade name, original title, device manufacturer, drug manufacturer, device trade name, keyword heading word, floating subheading word, candidate term word]	12199
6	1 or 5	12199
7	2 and 6	37
8	3 and 5	439
9	4 and 5	486
10	7 or 8 or 9	931

Medline 1946 to Feb week 5 via OVID 13 April 2024

Searches	Results	Type
1	microplastic pollution/ or microplastic/	7633
2	(women or woman).mp. [mp=title, book title, abstract, original title, name of substance word, subject heading word, floating sub-heading word, keyword heading word, organism supplementary concept word, protocol supplementary concept word, rare disease supplementary concept word,	1259835

	unique identifier, synonyms, population supplementary concept word, anatomy supplementary concept word]	
3	(people or person*).mp. [mp=title, book title, abstract, original title, name of substance word, subject heading word, floating sub-heading word, keyword heading word, organism supplementary concept word, protocol supplementary concept word, rare disease supplementary concept word, unique identifier, synonyms, population supplementary concept word, anatomy supplementary concept word]	1837851
4	(reproduction or reproductive).mp. [mp=title, book title, abstract, original title, name of substance word, subject heading word, floating sub-heading word, keyword heading word, organism supplementary concept word, protocol supplementary concept word, rare disease supplementary concept word, unique identifier, synonyms, population supplementary concept word, anatomy supplementary concept word]	320672
5	microplastic*.mp. [mp=title, book title, abstract, original title, name of substance word, subject heading word, floating sub-heading word, keyword heading word, organism supplementary concept word, protocol supplementary concept word, rare disease supplementary concept word, unique identifier, synonyms, population supplementary concept word, anatomy supplementary concept word]	9926
6	1 or 5	9926
7	2 and 6	17
8	3 and 5	332
9	4 and 5	407
10	7 or 8 or 9	737

Both databases deduplicated

	**Searches**	**Results**
1	microplastic pollution/ or microplastic/<br>Ovid MEDLINE(R) <1946 to February Week 5 2024><br>Embase <1974 to 2024 March 12>	17200<br>7633<br>9567

2	microplastic*.mp. [mp=ti, bt, ab, ot, nm, hw, fx, kf, ox, px, rx, ui, sy, ux, mx, tn, dm, mf, dv, dq]<br>Ovid MEDLINE(R) <1946 to February Week 5 2024><br>Embase <1974 to 2024 March 12>	22125<br>9926<br>12199
3	(women or woman).mp. [mp=ti, bt, ab, ot, nm, hw, fx, kf, ox, px, rx, ui, sy, ux, mx, tn, dm, mf, dv, dq]<br>Ovid MEDLINE(R) <1946 to February Week 5 2024><br>Embase <1974 to 2024 March 12>	3251249<br>1259835<br>1991414
4	(people or person*).mp. [mp=ti, bt, ab, ot, nm, hw, fx, kf, ox, px, rx, ui, sy, ux, mx, tn, dm, mf, dv, dq]<br>Ovid MEDLINE(R) <1946 to February Week 5 2024><br>Embase <1974 to 2024 March 12>	4501758<br>1837851<br>2663907
5	(reproduction or reproductive).mp. [mp=ti, bt, ab, ot, nm, hw, fx, kf, ox, px, rx, ui, sy, ux, mx, tn, dm, mf, dv, dq]<br>Ovid MEDLINE(R) <1946 to February Week 5 2024><br>Embase <1974 to 2024 March 12>	777368<br>320672<br>456696
6	1 or 2<br>Ovid MEDLINE(R) <1946 to February Week 5 2024><br>Embase <1974 to 2024 March 12>	22125<br>9926<br>12199
7	3 and 6<br>Ovid MEDLINE(R) <1946 to February Week 5 2024><br>Embase <1974 to 2024 March 12>	54<br>17<br>37
8	4 and 6<br>Ovid MEDLINE(R) <1946 to February Week 5 2024><br>Embase <1974 to 2024 March 12>	771<br>332<br>439
9	5 and 6<br>Ovid MEDLINE(R) <1946 to February Week 5 2024><br>Embase <1974 to 2024 March 12>	893<br>407<br>486

10	7 or 8 or 9<br>Ovid MEDLINE(R) <1946 to February Week 5 2024><br>Embase <1974 to 2024 March 12>	1668<br>737<br>931
11	remove duplicates from 10<br>Ovid MEDLINE(R) <1946 to February Week 5 2024><br>Embase <1974 to 2024 March 12>	1000<br>77<br>923

Supplementary material 2: Screening tool

Review question

'What is the impact of microplastics on women's reproductive health?'

Abstract and titles

1. Is the study an empirical study?
   *Reviews, editorials, letters, and studies without samples should be excluded

   Yes (Include) No (exclude)
2. Is the study available in English?

   Yes (Include) No (exclude)
3. Is the study focused on microplastics and human health?

   Yes (Include) No (exclude)
4. Is the study focused on the presence of microplastics in the environment or related to material development?

   Yes (Exclude) No (Include)

Full-text

5. Is the study focused on non-human reproductive health?

   Yes (Exclude) No (Include)

6. Is the study focused on women's reproductive health?
   *Consider including studies related to female fertility, uterus, ovaries, breasts, and placenta

   Yes (Include) No (exclude)

7. Are the microplastics 'naturally' present in the system, organ, or tissue being studied?
   *Exclude if MP were artificially exposed/added to tissues/organs

   Yes (Include) No (exclude)

Supplementary material 3: CASP checklist for descriptive/cross-sectional studies

Critical Appraisal Skills Programme (2024).

Questions

Q1: Did the study address a clearly focused issue?

Q2: Did the authors use an appropriate method to answer their question?

Q3: Were the subjects recruited in an acceptable way?

Q4: Were the measures accurately measured to reduce bias?

Q5: Were the data collected in a way that addressed the research issue?

Q6: Did the study have enough participants to minimise the play of chance?

Q7: How are the results presented and what is the main result?

Q8: Was the data analysis sufficiently rigorous?

Q9: Is there a clear statement of findings?

Q10: Can the results be applied to the local population?

Q11: How valuable is the research?
